# Mapping Aboveground Biomass of Four Typical Vegetation Types in the Poyang Lake Wetlands Based on Random Forest Modelling and Landsat Images

**DOI:** 10.3389/fpls.2019.01281

**Published:** 2019-10-16

**Authors:** Rongrong Wan, Peng Wang, Xiaolong Wang, Xin Yao, Xue Dai

**Affiliations:** ^1^Key Laboratory of Watershed Geographic Sciences, Nanjing Institute of Geography and Limnology, Chinese Academy of Sciences, Nanjing, China; ^2^College of Resources and Environment, University of Chinese Academy of Sciences, Beijing, China; ^3^School of Geographic Sciences, Nanjing University of Information Sciences and Technology, Nanjing, China

**Keywords:** aboveground biomass, wetland vegetation, random forest, Landsat image, Ramsar wetland, Poyang Lake

## Abstract

Wetland biomass is an important indicator of wetland ecosystem health. In this study, four dominant vegetation communities (*Carex cinerascens*, *Phalaris arundinacea*, *Artemisia selengensis*, and *Miscanthus sacchariflorus*) in the Poyang Lake wetland from 2010 to 2016 were classified from Landsat images using spectral information divergence (SID). We combined aboveground biomass (AGB) field measurements and remote sensing data to establish a suitable model for estimating wetland AGB in Poyang Lake, which is on the Ramsar Convention’s list of Wetlands of International Importance. The results showed that (1) overall, the classification accuracy for vegetation pixels across 5 years ranged from 59.1% to 73.7% and (2) the inter-annual and spatial variations in the AGB of the four vegetation types were clear. *C. cinerascens* had an average AGB density value of 1.28 kg m^−2^ in Poyang Lake from 2010 to 2016; *M. sacchariflorus *had the highest AGB density with an average value of 1.39 kg m^−2^; *A. selengensis* had almost the same level at 1.26 kg m^−2^; and *P. arundinacea* had the lowest AGB density at 0.64 kg m^−2^. This study provides useful experience for estimating carbon sequestration of vegetation in freshwater wetlands.

## Introduction

Wetland vegetation, as an important component of wetland ecosystems, plays an important role in maintaining ecosystem structure and function (Nilsson and Keddy, 1988; Pinay et al., 2002). The biomass of wetland vegetation is an important indicator to measure the health of wetland ecosystems and represents the relevant stage of wetland succession (Adam et al., 2010). Systematic studies on the wetland vegetation biomass of typical wetland ecosystems at different spatial and temporal scales not only can timely control the dynamic changes of wetland ecosystems but also can provide important parameters for the quantitative estimation of wetland ecosystem ecological assets and provide a scientific basis for the restoration, reconstruction, and management of wetland ecosystems (Parresol, 1999; Ursino, 2010; Klemas, 2013). Therefore, research on wetland biomass, especially aboveground biomass (AGB), is a hot research topic for researchers (Adam and Mutanga, 2012; Wan et al., 2018b).

In view of the diversity of wetland vegetation types, the dynamic changes in wetland biomass, and the inaccessibility of wetlands, the traditional biomass survey methods have obvious limitations in terms of labor costs, spatial comprehensiveness, and timeliness (Klemas, 2013). With the help of remote sensing technology, combined with field investigations in typical areas, it is possible to conduct research on long-term, dynamic, and fine spatial-scale observations of the vegetation biomass of typical wetland ecosystems (Shen et al., 2015; Zhu et al., 2015). Compared with the obvious stratification and zoning phenomena of dry land ecosystems, the plant unit of the wetland ecosystem environment usually presents a transient land–water interface, which makes the plant’s spectral characteristics and spatial distribution characteristics highly different, thus increasing the difficulty of large-scale AGB assessment of freshwater wetland vegetation (Adam and Mutanga, 2009; Zomer et al., 2009). Assessment of AGB for large wetlands is often challenging (Houlahan and Findlay, 2003). Studies of wetland biomass have focused mainly on AGB. Optical remote sensing, synthetic aperture radar (SAR), and light detection and ranging (LiDAR) are the three main methods for mapping wetland AGB. SAR and LiDAR are more suitable for the inversion of vegetation parameters with obvious structural characteristics, such as tall trees in a forest (Lefsky et al., 2002; Kasischke et al., 2003; Bortolot and Wynne, 2005; Englhart et al., 2011; Nie et al., 2017). For optical remote sensing, Landsat provides a trade-off of spatial, temporal, and spectral resolutions, which make it a good option for large-scale AGB modelling (Gasparri et al., 2010; Cutler et al., 2012; Main-Knorn et al., 2013; Zhu and Liu, 2015). Many vegetation indices have also been used widely in biomass estimation, resource surveys, vegetation dynamic monitoring, assessment of landscape structure and function, and global change research in recent years.

Poyang Lake is the largest freshwater lake in China. Its exposed floodplain in the dry seasons is one of the most important wetlands in the world, as recognized by the International Union for the Conservation of Nature (Ramsar Convention Secretariat, 2010). Accompanied by the fluctuating water level, the plant distribution in the Poyang Lake wetland is characterized by a typical concentric pattern along the elevation gradient from the lake to the shore (Wan et al., 2018a). Four main plant species are plentiful in the wetlands: *Carex cinerascens*, *Phalaris arundinacea*, *Miscanthus sacchariflorus*, and *Artemisia selengensis*. These species form three belts—bulrushes (*M. sacchariflorus* communities), sedges (*C. cinerascens* or *A. selengensis* communities), and sparse emergent vegetation (*P. arundinacea* communities)—that occur naturally along a moisture gradient from the higher lands to the lake shoreline (You et al., 2015; Wan et al., 2018a). Several researchers have attempted to estimate AGB in Poyang Lake wetlands using remote sensing approaches. Optical image data such as moderate-resolution imaging spectroradiometer (MODIS), Enhanced Thematic Mapper (ETM), and microwave remote sensing image data Envisat advanced SAR (ASAR) were used to fetch the estimations of the total AGB of Poyang Lake in spring (Li and Liu, 2002; Wang and Liao, 2010; Shen et al., 2015). Radar remote sensing is considered to have better accuracy in AGB prediction of *M. sacchariflorus* in the Poyang Lake wetlands, while the prediction accuracy of *C. cinerascens* is worse (Sang et al., 2014). Additionally, the long time series for estimation of AGB are mostly based on MODIS data applied to the power model of the enhanced vegetation index (EVI) to estimate the spatial distribution of vegetation AGB in the Poyang Lake National Nature Reserve (PLNNR) from 2000 to 2011 (Wu et al., 2015). Unlike other machine learning algorithms, random forests (RFs) (Breiman, 2001; Pal, 2005) can be used to predict target variables based on high-dimensional data without feature selection. Some studies have also shown that RF has a high accuracy in predicting AGB (Mutanga et al., 2012; Byrd et al., 2018). Thus, in this study, the ensemble method RF was used to estimate AGB in the Poyang Lake wetlands from 2010 to 2016, aimed at quantitatively estimating the AGB of the four main vegetation communities and their distribution and various characteristics.

## Materials and Methods

### Study Area

Poyang Lake is located at 115°47ʹ–116°45ʹE and 28°22ʹ–29°45ʹN of Jiangxi Province and on the south bank of the middle and lower reaches of the Yangtze River. Its north–south length is approximately 170 km, its east–west average width is 16.9 km, and its maximum width is 74 km. Poyang Lake mainly collects incoming water from the Ganjiang, Fuhe, Xinjiang, Raohe, and Xiushui rivers. After regulation and storage, it flows into and interacts with the Yangtze River from Hukou. Poyang Lake is in the subtropical humid monsoon climate, with an annual average temperature of approximately 17°C. Rainfall is abundant, averaging approximately 1,600 mm annually. From July to August, the water level of the lake reaches the maximum for the year, and the area of the lake can reach 4,070 km^2^. During the dry season from December to January of the next year, the water level of the lake decreases greatly, and the area of the lake can reach a minimum of 146 km^2^. In this special hydrological and topographic environment, it has evolved into a unique water area and wetland ecosystem. The Poyang Lake wetland, in the littoral zone of Poyang Lake, was one of the first to be included in the Ramsar Convention’s list of Wetlands of International Importance (Ramsar Convention Secretariat, 2010).

### Field Surveying and Data Collection

**Figure 1:**
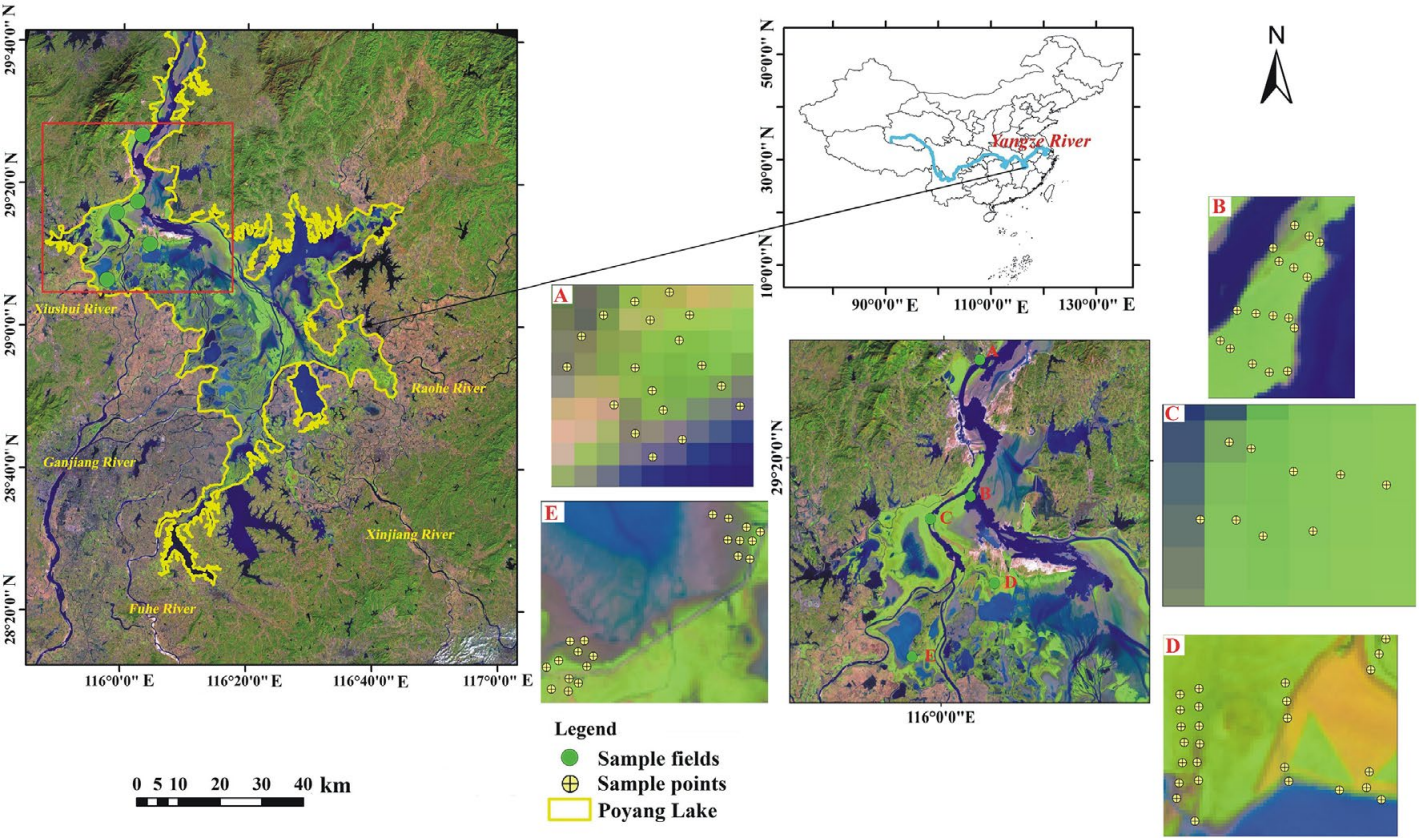
Location of Poyang Lake and the distribution of field sampling sits in 2016. **(A)** Xingzi littoral land; **(B)** Ganjang river delta; **(C)** Sidu Island; **(D)** Dachahu sub-lake; **(E)** Dahuchi sub-lake.

**Figure 2:**
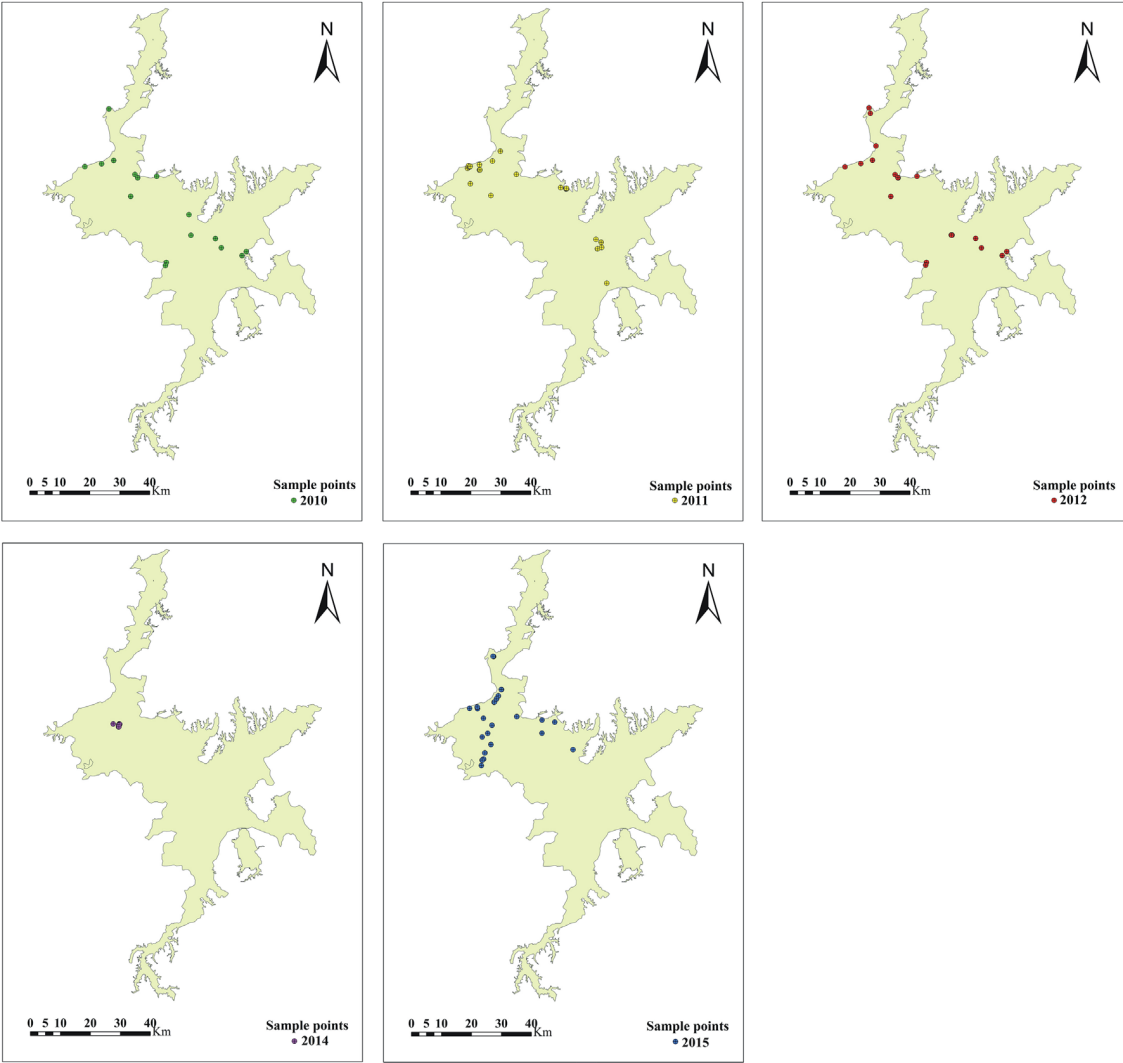
Spatial distribution of sample points for model validation in 2010, 2011, 2012, 2014, and 2015.

To carry out the inversion of AGBs in the Poyang Lake wetland, three types of basic data were collected, including remote sensing image data, topographic data digital elevation model (DEM), and field survey data. In terms of remote sensing image data, we collected four Landsat 8 and three Landsat 7 scenes covering the whole area of Poyang Lake from 2010 to 2016 with the imaging time of October to December. The remote sensing images from October to December were selected because *A. selengensis* and *C. cinerascens* were still in the peak growing season during this period, while *M. sacchariflorus* had entered the wilting period, which could maximize the spectral characteristics of different vegetation communities. The data are from the United States Geological Survey (USGS) (http://earthexplorer.usgs.gov/), and the specific image time is shown in **Table 1**. The cloud content of all images is less than 10%, which reduces the impact of other environmental factors on the remote sensing interpretation of vegetation. The DEM data are a 1:10,000 topographic map provided by the Jiangxi Provincial Hydrological Bureau. For field survey data, we conducted field sampling and actual positioning surveys in Poyang Lake in December 2016. Five typical beaches of Poyang Lake wetland ((A) Xingzi littoral land, (B) Ganjiang River delta, (C) Sidu Island, (D) Dachahu sub-lake, and (E) Dahuchi sub-lake; **Figure 1**, **Table 2**) were selected with 123 sample sites covering the four main vegetation types of Poyang Lake. The elevation of these points increased from water to shore, and the sample size is 1 m * 1 m. The geographical coordinates, main vegetation types, and the percentage and average height of vegetation cover at each sample site were recorded. Vegetation above the surface in the sample box was harvested, and the fresh weight was measured. To validate the accuracy of classification and AGB inversion in different years, we collected vegetation type and AGB data from 142 field points sampled by our work team in autumn 2010, 2011, 2012, 2014, and 2015 (**Figure 2**).

**Table 1:** The timing of remote sensing of images used in this study.

Time	Data Source	Time	Data Source
Nov 6, 2010	Landsat 7	Oct 21, 2013	Landsat 8
Oct 8, 2011	Landsat 7	Oct 24, 2014	Landsat 8
Oct 26, 2012	Landsat 7	Oct 11, 2015	Landsat 8
		Dec 16, 2016	Landsat 8

**Table 2:** The number of sample sites in every sample field in 2016.

Sample field	*C. cinerascen*	*P. arundinace*	*A. selengensis*	*M. saccharifloruss*	Total
(A) Xingzi littoral land	16	2	3	3	24
(B) Ganjiang River delta	5	4	6	2	17
(C) Sidu Island	5	2	3	2	12
(D) Dachahu sub-lake	27	7	2	7	43
(E) Dahuchi sub-lake	11	6	3	7	27
Total	64	21	17	21	123

The locations of sample fields are shown in **Figure 1**.

### Data Preprocessing and Preparation

**Figure 3:**
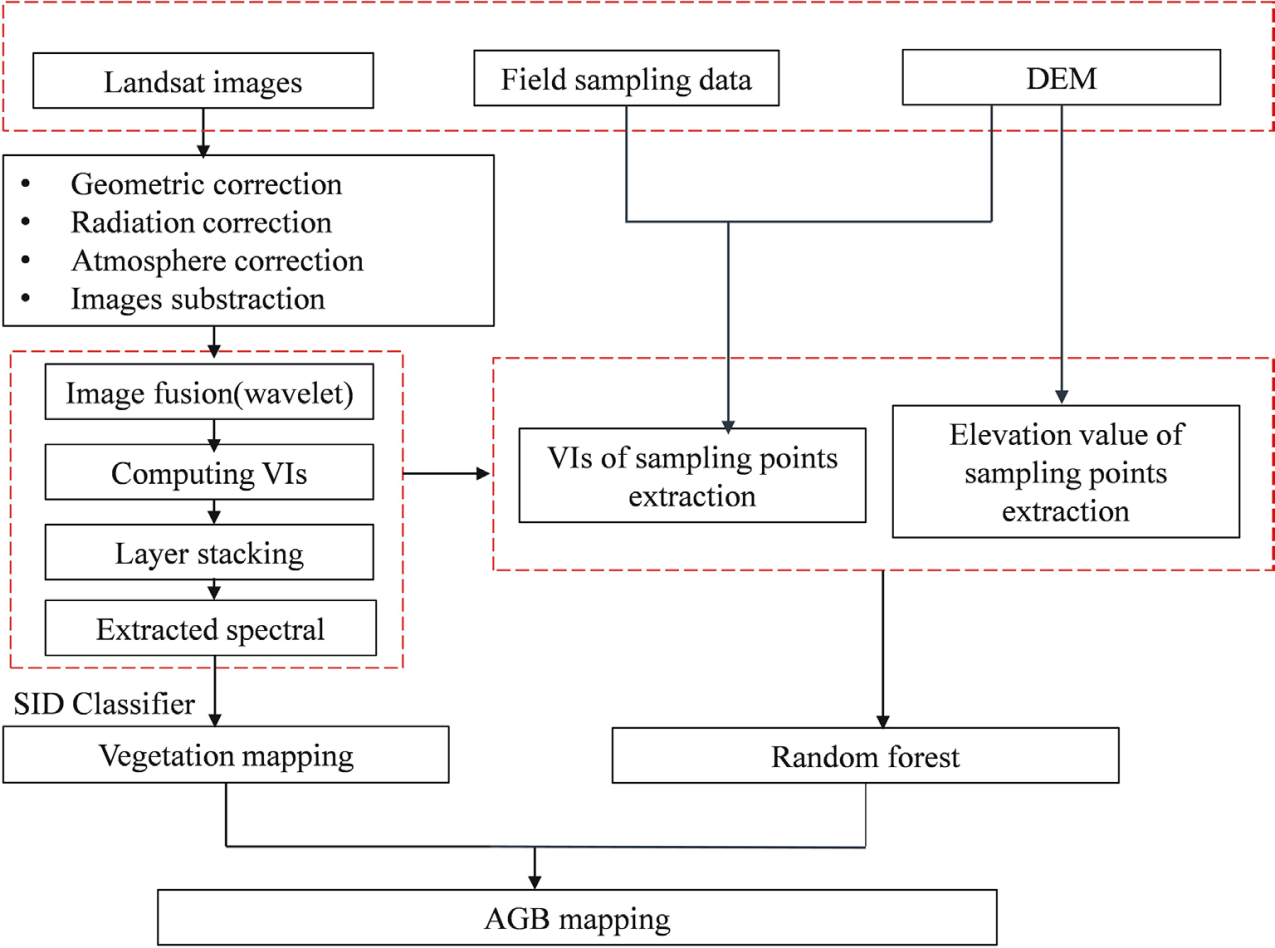
Flowchart used to map AGB in Poyang Lake using Landsat images.

Landsat 8 OLI_TIRS features 11 bands: band 1 (0.43–0.45 μm), band 2 (0.45–0.51 μm), band 3 (0.53–0.59 μm), band 4 (0.64–0.67 μm), band 5 (0.85–0.88 μm), band 6 (1.57–1.65 μm), band 7 (2.11–2.29 μm), and band 9 (1.36–1.38 μm) with spatial resolutions of 30 m; band 8 (0.50–0.68 μm) with a spatial resolution of 15 m; and band 10 (10.6–11.19 μm) and band 11 (11.5–12.51 μm) with spatial resolutions of 100 m. Landsat 7 ETM+ SLC-off has eight bands: band 1 (0.45–0.52 μm), band 2 (0.52–0.60 μm), band 3 (0.63–0.69 μm), band 4 (0.77–0.90 μm), and band 5 (1.55–1.75 μm) with spatial resolutions of 30 m; band 6 (10.40–12.50 μm) with a spatial resolution of 60 m; and band 8 (0.52–0.90 μm) with a spatial resolution of 15 m. It must be noted that Landsat 7 ETM+ encountered a malfunction on May 31, 2003, that led to some images overlapping and the loss of approximately 25% of our data; therefore, the images obtained after this date were restored by the neighborhood similar pixel interpolator (NSPI) proposed by Chen et al. (2011). Image preprocessing, including geometric correction, radiometric correction, atmospheric correction, and spatial subset selection, was finished in ENVI 5.2. We also utilized a panchromatic band (0.500–0.680 μm) with a spatial resolution of 15 m to create an image fusion with multispectral bands and a spatial resolution of 30 m to produce a multispectral image with a spatial resolution of 15 m by employing a method called wavelet fusion (Fanelli et al., 2001), which was aimed at improving the classification accuracy for all four vegetation types in Poyang Lake wetland. Six bands from the Kauth–Thomas transformation (Kauth and Thomas, 1976) and normal difference vegetation index (NDVI), soil-adjusted vegetation index (SAVI), and EVI were added to the fused images to generate layer-stacked images. Then, the layer-stacked image from December 16, 2016, was chosen for extraction of all 123 spectral sampling sites, according to the geographical coordinates recorded by Global Positioning System (GPS), in preparation for classification of the four Landsat 8 images. For the three Landsat 7 images, 82 sampling points covered the four dominant vegetation types in the Poyang Lake wetlands that were generated from the vegetation map of PLNNR in the autumn of 2010 compiled by Nanchang University (Jin et al., 2016). Then, the spectra of these 82 sampling points were used to classify the three layer-stacked 15-band Landsat 7 images. The proposed methods are briefly explained in the flowchart (**Figure 3**).

### Methods

#### Mapping Dominant Vegetation Types

All image classification was undertaken using the spectral information divergence (SID) classifier (Chang, 1999) in conjunction with the spectral library created from sampling points. Constrained by the band number and the spatial resolution of the layer-stacked images, only seven types of objects, including four vegetation communities *(C. cinerascens*, *P. arundinacea*, *A. selengensis*, and *M. sacchariflorus*) and three land types (water body, mudflat, and bare land), were plotted on the maps. We carried out many classification trials to determine an appropriate parameter—the maximum divergence threshold—with which to distinguish all vegetation classes. Finally, we found that 0.06 was the most effective threshold value.

#### AGB Modelling Methods

In this study, we evaluated the effectiveness of the RF model in estimating AGB in Poyang Lake. Then, we utilized the classification images and RF model to map the four dominant vegetation communities in autumn from 2010 to 2016. Researchers have proposed modified vegetation indices (VIs), including SAVI (Huete, 1988), modified SAVI (MSAVI) (Qi et al., 1994), and EVI (Liu and Huete, 1995), to overcome the weaknesses of NDVI; namely, that it is easily affected by the atmosphere, soil composition, and heavy saturation in dense vegetation. We selected nine variables as predictors of the RF model, including NDVI, SAVI, EVI, B3 (red band), B4 (near infrared (NIR) band), greenness (the second band of the Kauth–Thomas transformation), B6 (SWIR1, short-wave infrared band 1), B7 (SWIR2, short-wave infrared band 2), and elevation. The reasons for selecting these predictors to train the RF are as follows: NDVI is the most commonly used vegetation index for identifying vegetation (Defries and Townshend, 1994; Jia et al., 2014). In wetland areas, SAVI, Greenness, and EVI can effectively remove the influence of soil background and enhance the response of the vegetation index to wetland vegetation (Jiang et al., 2008; Ren et al., 2018). The red band and NIR band for Landsat images have a better optical response to chlorophyll in vegetation (Clevers et al., 2008). The spectral absorption rate of vegetation in the red band is higher, but the reflectivity of the NIR band is higher; these two bands have a good response to vegetation biomass. The thermodynamic properties of vegetation and water in wetland areas are different, so including SWIR1 and SWIR2 is helpful to identify vegetation and water, and the contrast between different vegetation types is also stronger (Mayer and Scribner, 2002). Four main dominated wetland vegetation communities in the Poyang Lake wetland are distributed along elevation gradient from the lake to the shore. Taking elevation (representing moisture condition) as a predicting factor may increase the accuracy of the RF model. All the variables’ values for all 123 sampling sites were extracted according to their geographic coordinates.

**Figure 4:**
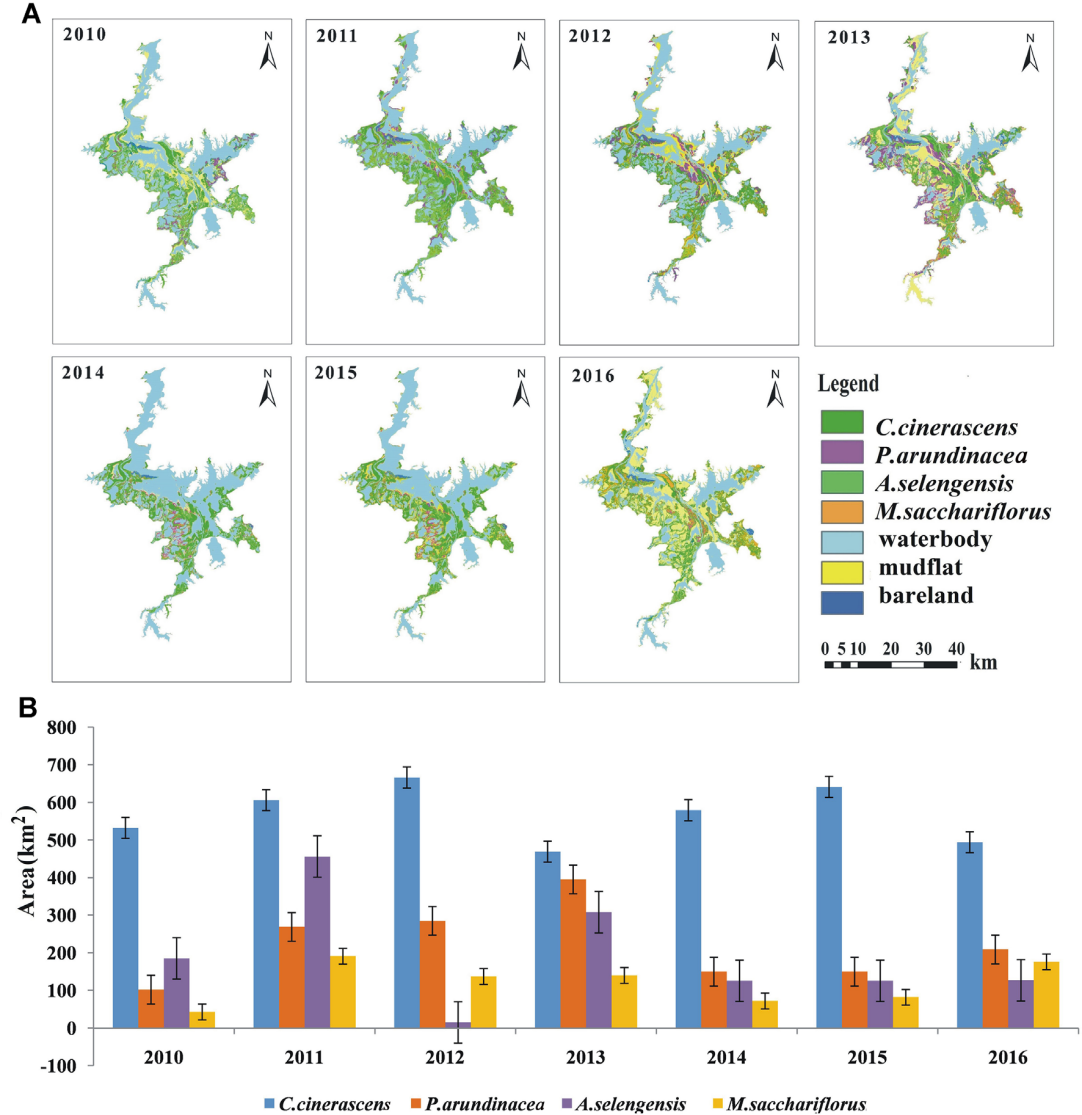
Distribution of the main four vegetation types **(A)** and area statistics **(B)** from 2010 to 2016.

RF is an important ensemble learning method based on bagging that can be used for classification, regression, and other techniques. The structure of the RF model for estimating the AGB of Poyang Lake is shown in **Figure 4**. On the basis of root mean square error (RMSE), *R* ^2 ^(determination coefficient), and mean absolute error (MAE), we evaluated the accuracy of the RF model in predicting AGB on the training set and test set. RMSE (Equation 1) is a standard metric for measuring the discrepancies between the simulated AGB value and the actual AGB value; however, it is easily influenced by outliers (Chai and Draxler, 2014). Therefore, MAE (Equation 2) is suggested for use with RMSE to determine the variation of errors in the model (Bui et al., 2016). *R* ^2^ (Equation 3) is a measure of the proportion of variance of a predicted outcome. RMSE and MAE values close to 0 and *R* ^2^ values close to 1 indicate that the model is an accurate predictor. Refer to Equations 1, 2, and 3 for the calculation of RMSE, MAE, and *R* ^2^, respectively. Three parameters must be optimized in this model: (1) *N*, the number of regression trees grown based on a bootstrap sample of the observations; (2) *mtry*, the number of predictors tested at each node; and (3) *node size*, the minimum size of the terminal nodes of the trees. For the parameter *node size*, the accuracy of the RF models on the training dataset was compared based on RMSE when *node size* was set from 1 to 4, so that the best node size can be obtained. For the determination of the number of trees (*N*) for RF, we increase the number by two trees in turn from *N* = 60 until the number of trees is 800, so 371 models with different *N* are tried. At the same time, the values of RMSE, *R* ^2^, and MAE of AGB for these models with different tree numbers are calculated using five-fold cross-validation. Thus, the performance of RF in predicting the AGB of Poyang Lake was evaluated comprehensively, and the best *N* was obtained. In this study, we implemented the RF modelling method through packages in Python: scikit-learn (http://scikit-learn.org/stable/ ) (Pedregosa et al., 2011).

(1) $$\mathrm{RMSE}=\sqrt{\sum_{i=1}^{n}\frac{{\left(y_{\mathrm{is}}-y_{\mathrm{it}}\right)}^{2}}{n}}$$

(2) $$\mathrm{MAE}=\frac{\sum_{i=1}^{n}\left|y_{\mathrm{is}}-y_{\mathrm{it}}\right|}{n}$$

(3) $$R^{2}=1-\frac{\sum_{i=1}^{n}{\left(y_{\mathrm{is}}-y_{\mathrm{it}}\right)}^{2}}{\sum_{1=1}^{n}{\left(y_{\mathrm{is}}-{\bar{y}}_{\mathrm{is}}\right)}^{2}}$$

where *y_is_* is the *i*th simulated AGB value, *y_it_* is the *i*th real AGB value among the tested sample points, ${\bar{y}}_{is}$ is the average simulated AGB for all the tested sample points, and *n* is the size of the tested samples.

## Results

### Image Classification

To explore the classification accuracy, we used data from 142 field sampling points in autumn 2010, 2011, 2012, 2014, and 2015 for validation. The specific results are shown in **Table 3**. The classification accuracy over the 5 years ranged from 59.1% to 73.7%. The 5-year average classification accuracy for the four kinds of vegetation is 72.14% for *Carex cinerascens*, 64.26% for *Phalaris arundinacea*, 49.38% for *Artemisia selengensis*, and 71.68% for *Miscanthus sacchariflorus*. We found that *C. cinerascens *and *M. sacchariflorus* have higher classification accuracy (approximately 70%), while *A. selengensis* had relatively low classification accuracy (approximately 50%). According to a field survey, we found that in bottomlands, *A. selengensis* tended to grow together with *P. arundinacea*, which led to lower classification accuracy. *C. cinerascens* is the most widely distributed vegetation in the wetlands of Poyang Lake. It has the characteristics of concentrated and patchy distribution. In autumn, many *M. sacchariflorus* plants have withered, and the spectral characteristics are quite different from those of other vegetation. Therefore, these two vegetation communities are easy to identify. Although the amount of sample points is relatively small, the results are acceptable given that complicated environmental conditions such as changeable water level and variable atmospheric conditions.

**Table 3:** Summary of producer’s accuracy for the four dominant vegetation communities of classification results based on field survey data in 2010, 2011, 2012, 2014 and 2015.

Land cover type	2010		2011		2012		2014		2015	
	NOSP	PA(%)	NOSP	PA(%)	NOSP	PA(%)	NOSP	PA(%)	NOSP	PA(%)
*C. cinerascens*	7	71.4	3	100	7	57.1	10	60	18	72.2
*P. arundinacea*	5	60	4	75	5	80	2	50	16	56.3
*A. selengensis*	3	33.3	8	50	4	50	6	50	11	63.6
*M. sacchariflorus*	4	75	9	66.7	4	75	4	75	12	66.7
Total	19	73.7	24	66.7	20	65	22	59.1	57	64.9
NOSP refer to number of sampling points, PA refer to producer’s accuracy.										

**Figure 4** shows the distribution and statistical results of all four vegetation classes from 2010 to 2016. From 2010 to 2016, *C. cinerascens* had the largest distribution, with an average area of 569 km^2^. The distributions of *P. arundinacea* and *A. selengensis *were 226 and 200 km^2^, respectively. The smallest was *M. sacchariflorus* with a distribution area of only 120 km^2^. In 2011 and 2010, Poyang Lake had the largest and smallest vegetation coverages of 1,522 and 861 km^2^, respectively. Generally, the coverage of each of the vegetation types is always changing as a result of water level fluctuations. The complicated hydrological and ecological processes of the Poyang Lake wetlands greatly influence the development of vegetation in the bottomlands of the lake.

### Accuracy of the RF Model for Estimating AGB in the Poyang Lake Wetland

**Figure 5:**
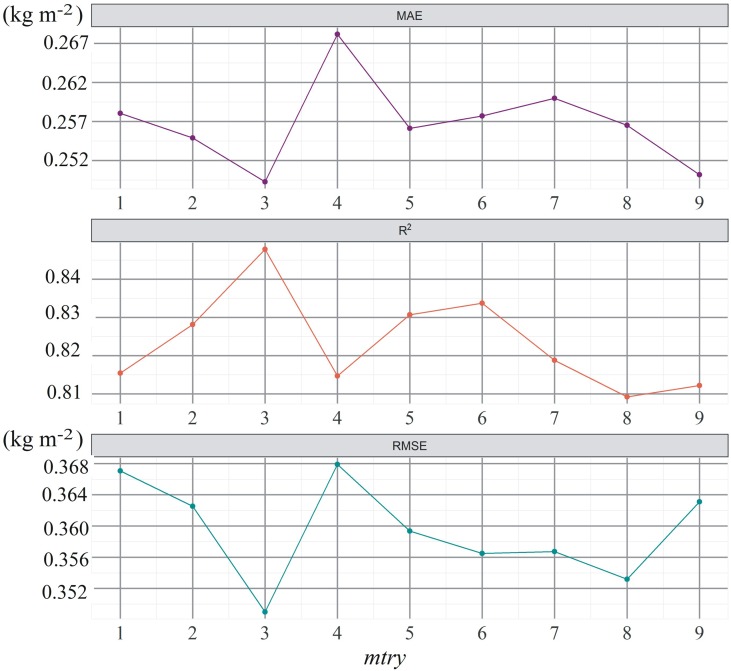
Prediction accuracy of the RF model on training data set when *mtry* varies from 1 to 9.

**Figure 6:**
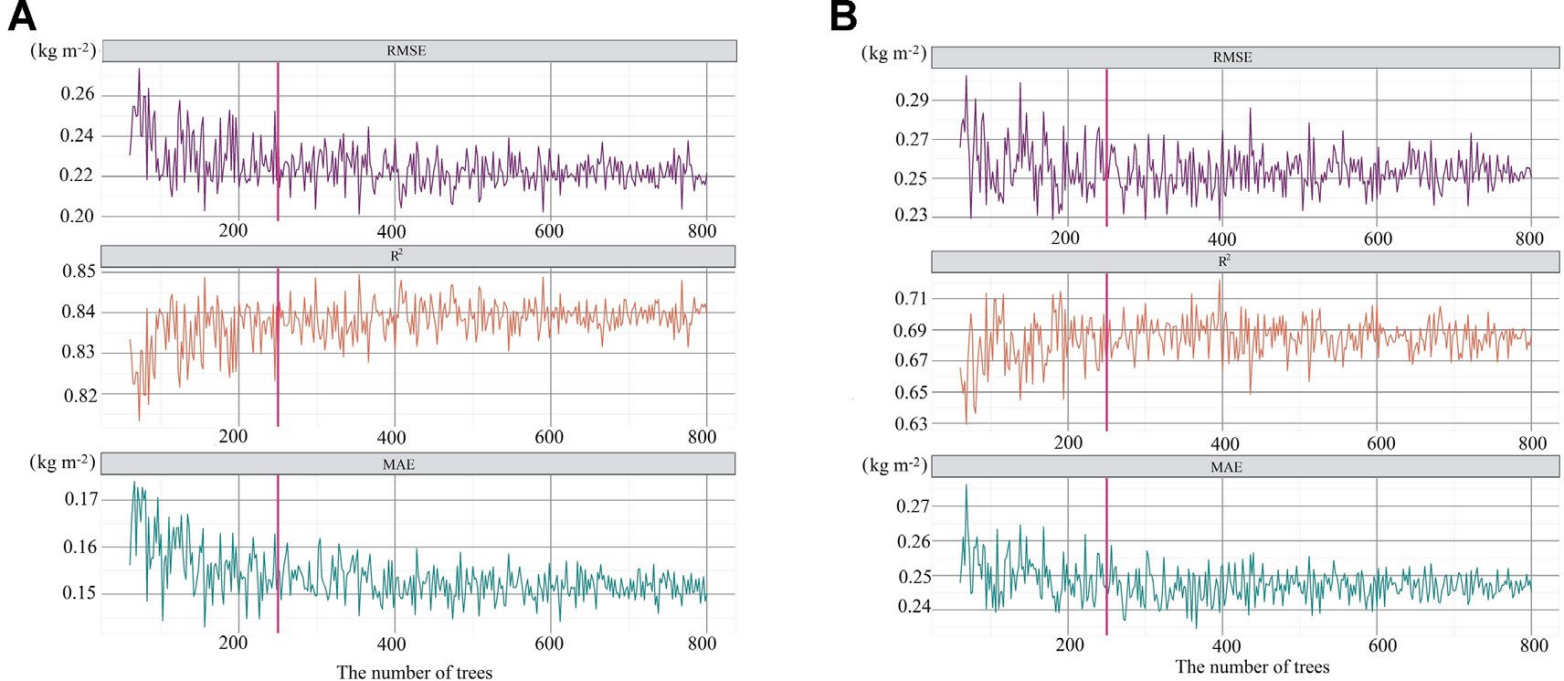
Average accuracy of five-fold cross-validation of RF Model in training samples **(A)** and testing samples **(B)**.

**Figure 7:**
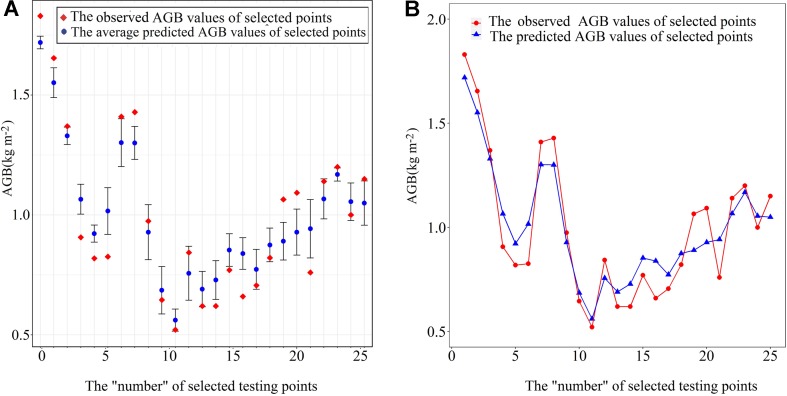
Comparison of predicted with error bar in **(A)** and without error bar in **(B)** and actual values of AGB on RF Model validation sample set when *N*=390.

The average and standard deviation values of AGB of four vegetation types were obtained by statistical analysis of sample points (**Table 4**). The AGB of four vegetation types was ranked from low to high as *P. arundinacea*, *C. cinerascens*, *A. selengensis*, and *M. sacchariflorus*. The change in AGB in *M. sacchariflorus* communities was obvious, which might be related to the different wilting degree of *M. sacchariflorus* in some sample sites. We found that the AGB prediction accuracy of RF on training set mainly used for calibration of model parameters is higher and more robust when the *node size* is 2. **Figure 5** shows that when *node size* is 2, the accuracy of the RF model after five-fold cross-validation varies along with *mtry*. It can be found that when *mtry* is 3, the model has the highest accuracy on the training set. **Figure 6A** shows the values of RMSE, *R* ^2^, and MAE for RF models with different numbers of trees after five-fold cross-validation in the training dataset (*mtry* = 3, *node size* = 2). RMSE ranged from 0.20 to 0.28 kg m^−2^, *R* ^2^ ranged from 0.80 to 0.85, and MAE ranged from 0.13 to 0.18 kg m^−2^. **Figure 6B** shows the values of RMSE, *R* ^2^, and MAE for RF models with different numbers of trees after five-fold cross-validation in the testing dataset. RMSE ranged from 0.23 to 0.30 kg m^−2^, *R* ^2^ ranged from 0.63 to 0.72, and MAE ranged from 0.23 to 0.28 kg m^−2^. When the number of trees is 250, the values of the three indicators in the training dataset and testing dataset tend to be relatively stable, indicating that the comprehensive performance of the model has not been significantly improved with the change in the number of *N*. The average value for the three criteria of RF algorithms is shown in **Table 5**. Compared with the performance of RF on training datasets, the prediction accuracy of AGB on the test datasets is lower. We think the average RMSE value of 0.26 kg m^−2 ^is satisfactory given that the sampling points are not sufficient. When *N* = 390, the model has the lowest RMSE value (0.23 kg m^−2^) and the highest *R* ^2^ (0.72) in the test set, so the model has the best prediction accuracy for AGB (**Figure 6**). And then 20% of the samples were randomly selected as validating points, and the AGB values of these 20 percentage points were predicted based on five models calibrated by five-fold cross-validation (*N* = 390, *mtry* = 3, *node size* = 2). The results are shown in **Figure 7**. In summary, we concluded that RF was a satisfactory model for use in predicting AGB in the Poyang Lake wetlands.

**Table 4:** Statistical characteristics of AGB of four vegetation communities of field sampling in 2016 December.

	*C. cinerascens*	*P. arundinacea*	*A. selengensis*	*M. sacchariflorus*
Average	1.09	0.88	1.42	1.70
SD	0.54	0.38	0.56	0.95

**Table 5:** The average RMSE (kg m^-2^), R^2^ and MAE (kg m^-2^) values for RF models with different trees to estimating AGB in the training and testing datasets.

Model	Training dataset			Testing dataset		
	RMSE	R^2^	MAE	RMSE	R^2^	MAE
RF	0.23	0.84	0.15	0.26	0.68	0.25

### Predicting the AGB of Various Vegetation Communities

**Figure 8:**
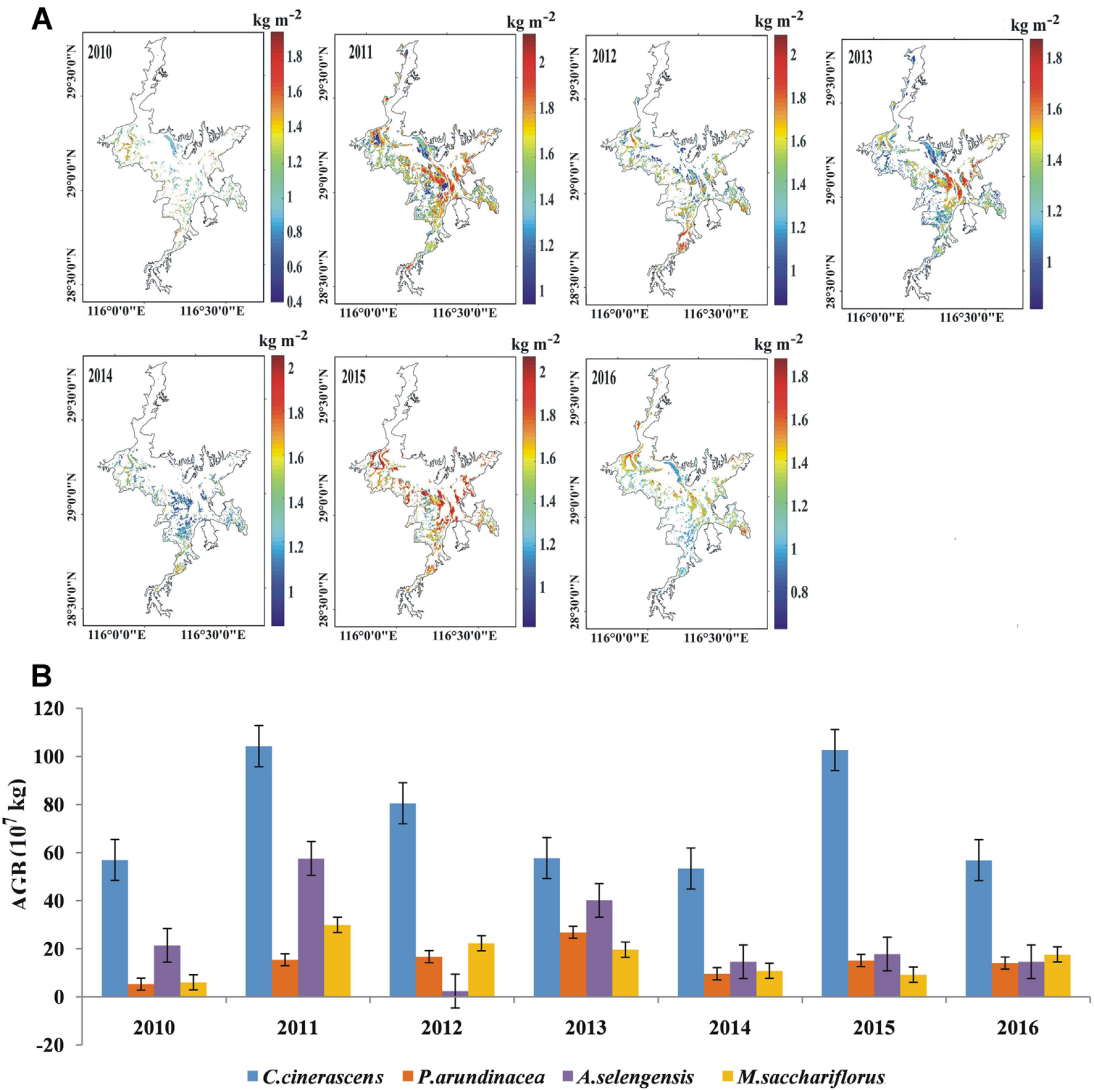
AGB density distribution **(A)** and total AGB statistics **(B)** in Poyang Lake wetland in autumn from 2010 to 2016.

We utilized the trained RF (*N* = 390) to explore the AGB distribution in Poyang Lake from 2010 to 2016 (**Figure 8**). The average annual total AGB in the Poyang Lake wetland in the past 7 years is approximately 1.28 × 10^9^ kg. To validate the accuracy of AGB inversion in different years, we used the measured AGB of the 142 field sample points in autumn 2010, 2011, 2012, 2014, and 2015 to calculate RMSEs (**Table 6**). We found that the RMSEs of 5 years ranged from 0.41 to 0.52 kg m^−2^. We found that the AGB distribution was abundant in 2011, 2012, 2013, and 2015, especially in 2011 and 2015, all over the lake; however, 2010, 2014, and 2016 were relatively deficient (**Figure 8**). Generally, a higher-than-average AGB value of more than 1.2 kg m^−2^ occurred in the northwest and southeast parts of Poyang Lake, while the southwest and northeast experienced lower AGB values, except in 2011 and 2015. **Figure 8** showed the total AGB statistics from 2010 to 2016 for the most common vegetation communities. *C. cinerascens* had the highest total value of 1.28 kg m^−2^ (**Table 7**) because it had the largest distribution area in Poyang Lake; *A. selengensis* and *M. sacchariflorus* had the second- and third-largest biomass, respectively; and *P. arundinacea* had the lowest biomass value. *M. sacchariflorus* had the highest AGB density, with an average value of 1.39 kg m^−2^; *C. cinerascens* and *A. selengensis *were almost at the same level, with 1.26 and 1.28 kg m^−2^, respectively; and *P. arundinacea* had the lowest at 0.64 kg m^−2 ^(**Table 7**).

**Table 6:** Validation accuracy for AGB inversion in 2010,2011,2012,2014 and 2015.

	2010	2011	2012	2014	2015
Numbers of sampling points	19	24	20	22	57
RMSE(kg m^-2^)	0.52	0.47	0.52	0.49	0.41

**Table 7:** The four most common vegetation communities’ biomass densities (kg m^-2^) in Poyang Lake wetland from 2010 to 2016.

Year	*C. cinerascens*	*P. arundinacea*	*A. selengensis*	*M. sacchariflorus*	Ave.
2010	1.07	0.52	1.16	1.48	1.06
2011	1.72	0.57	1.26	1.56	1.28
2012	1.22	0.56	1.73	1.65	1.29
2013	1.25	0.66	1.30	1.40	1.15
2014	0.93	0.53	0.77	1.50	0.93
2015	1.63	0.98	1.42	1.16	1.30
2016	1.15	0.67	1.16	1.00	0.99
Ave.	1.28	0.64	1.26	1.39	

## Discussion

### Distribution of AGB in Poyang Lake Wetlands and Its Causes

The change in the inter-annual and intra-annual hydrological variations of Poyang Lake are the main factors leading to the inter-annual change in wetland AGB. The annual water level fluctuations are relatively large, with the difference between the highest and lowest water levels of Poyang Lake exceeding 10 m (Dai et al., 2015; Wan et al., 2018c). The imaging time for the selected remote sensing images is not entirely the same. The fluctuation of the water level leads to a change in the bare beach area that then affects the AGB in the Poyang Lake wetland. The difference in spatial distribution of AGB in the same period may be related to the tolerance of different vegetation communities to hydrological conditions. Undoubtedly, fluctuations in water conditions such as water level, water table level, water depth, soil water content, and percentage of inundated days per year in the wetland affect the growth and distribution of vegetation (Stromberg et al., 1996; Katz et al., 2009; Toogood and Joyce, 2009). Given its unique geographical location and characteristics of river–lake exchange, the seasonal variation in water conditions in the Poyang Lake wetlands is very significant. *Carex cinerascens* can tolerate a wide variety of hydrological processes, so *C. cinerascens* could be widely distributed in the Poyang Lake wetland, while *Miscanthus sacchariflorus* only adapts to a relatively small range of hydrological conditions (Zhang et al., 2012; Wan et al., 2018a; Dai et al., 2019). An excessively high water level does not benefit the growth of *M. sacchariflorus*, which indicates that *M. sacchariflorus* grows on the beach at higher altitudes.

Our results show that the low-AGB areas of the Poyang Lake wetland are generally concentrated in the center of sub-lakes or dish-shaped bottomlands. The frequency of flooding in these areas is higher than that in high bottomlands. *Phalaris arundinacea*, with its flat leaf blade and 30 to 50cm stem height, grows in the easily flooded low-lying bottomlands and has strong adaptability to higher soil moisture content, which makes a lower AGB occur in these places. *Artemisia selengensis* and *M. sacchariflorus* are located in the highlands of the Poyang Lake wetland at a 14 to 17m elevation. They are less likely to experience flooding, to grow taller, or to have thicker stalks. Their AGB density was higher than that of *P. arundinacea* and *C. cinerascens*. Therefore, there are higher AGBs in some high-elevation alluvial deltas, such as the Ganjiang River delta. *C. cinerascens*, the most common vegetation category in Poyang Lake, is often found in the areas between 13 and 15 m, where water has no adverse effects for most of the year, except during the flood season, which allows them to have a higher AGB density value. From October to December, with the gradual decline in water level, *C. cinerascens* communities are exposed. At this time, vegetation communities such as those of *A. selengensis* and *M. sacchariflorus* experienced blooming or withering stages, which caused the AGB density of *M. sacchariflorus* in the dry season to be lower than that of *M. sacchariflorus* in the growing season.

### Analysis of the Accuracy and Uncertainty of the Study

This study represents the first time that a landscape-scale remote sensing model of AGB for seasonal lake wetlands in floodplain areas has been produced based on RF algorithms and Landsat images. First, we chose the images whose imaging time is similar to that of the dry season of the lake, which ensures that the hydrological environment of the wetland of Poyang Lake is relatively similar at the imaging time of each year. Similar hydrological environments make the spectral characteristics of the vegetation samples collected in 2016 consistent with those of other years to the greatest extent possible. In addition, in this period, the beach of Poyang Lake, is exposed, and the distribution area of vegetation is the largest in autumn and winter. The daily water levels of Poyang Lake, which were 11.65, 11.53, 11.77, 9.43, 11.47, 13.38, and 9.24 m in 2010–2016, respectively, corresponded to the times of image acquisition. From the results of our classification maps, the dynamic change in the exposed area of the beach landscape is consistent with the fluctuation of the water level. In 2010, 2011, 2012, 2014, and 2015, the daily water levels are close, and the size of the exposed beach landscape is similar. The same is true for 2013 and 2016. Second, the validation sampling period for AGB in other years is the same as in 2016, which is October to December, around the dry season of Poyang Lake. We applied the training model for 2016 to AGB inversion in 2010–2015 (except 2013), and the RMSE values were from 0.41 to 0.52 kg m^−2^. The results were acceptable, although the accuracy was slightly lower than that in 2016.

To our knowledge, there are very few works focused on estimating the total AGB of the Poyang Lake wetlands from the perspective of the vegetation community in a withered period on a multi-year timescale. Our results show that the total AGB of Poyang Lake in autumn or winter from 2010 to 2016 was 0.90 × 10^9^, 2.07 × 10^9^, 1.22 × 10^9^, 1.44 × 10^9^, 0.88 × 10^9^, 1.45 × 10^9^, and 1.03 × 10^9^ kg, respectively. The average total AGB for 7 years was 1.28 × 10^9^ and 0.27 × 10^9^ kg higher than the result of Dong et al. (2008). It is noteworthy that our models have a better accuracy than that work (RMSE: 0.4 kg m^−2^). More research on the AGB estimation of the vegetation growth period in Poyang Lake wetlands has been performed. Wang and Liao, (2010) estimated the AGB of the Poyang Lake wetland in April 2007 using Landsat TM and Envisat ASAR data and concluded that the total AGB of the whole Poyang Lake was approximately 2.1 × 10^9^ kg. Li and Liu (2002) estimated the total biomass of Poyang Lake wetlands in April 2000 using a simple linear regression model, which was approximately 3.8 × 10^9^ kg. Their estimations are much higher than our results, mainly because they focus on the vegetation AGB of Poyang Lake wetlands in the vegetation growth period. Given the insignificant spectral difference in the dominant vegetation types of Poyang Lake in spring, and the oversaturation of the biomass response to the vegetation index, we hold that it is not appropriate to identify the distribution of vegetation types and that it will not result in an accurate AGB estimation in this period. Moreover, these works were all attempting to make an overall AGB estimation for the Poyang Lake wetland at a single time point, not long-term specifications for the AGB of four dominant types of plants in the Poyang Lake wetland. Therefore, the results are undoubtedly highly uncertain and difficult to use to show a trend in AGB over a long period of time. A power model of the EVI was used to estimate the spatial distribution of vegetation AGB from 2000 to 2011in the PLNNR, located in the north of the Poyang Lake wetland (Wu et al., 2015). The overall fitting accuracy is 91.7%, based on MODIS long time series data. However, MODIS data are limited by spatial resolution, so it is difficult to identify the spatial characteristics of the dominant vegetation types or estimate the biomass of different vegetation types in Poyang Lake.

The uncertainty in accuracy is mainly due to several reasons. Differences in atmospheric conditions and lake water levels may lead to errors in vegetation classification and AGB inversion. The atmospheric conditions during the imaging period will affect the grey value of the pixels, resulting in the phenomenon of homologous vegetation with different spectra. We tried to reduce errors by selecting images from around the dry season and performing field samplings in the period from late October to late November. However, incomplete temporal inconsistency of data still exists. Subtle changes in the spectral characteristics of vegetation from October to December and mixed vegetation communities in some areas make it difficult to identify vegetation accurately. In autumn, emergent vegetation such as *M. sacchariflorus* enters the heading stage from October to November, and the aboveground stems wither and die in late December. Sparse emergent vegetation such as *P. arundinacea* and *A. selengensis* communities begin to wither and die from October to November. The *C. cinerascens *community has a longer lifetime. It has developed the distinctive feature of initiating growth twice within a year, in spring and autumn. In September, the water level recedes, and the *C. cinerascens* community initiates growth as the sites are eventually exposed. The biomass reaches a maximum during October and November. Although the AGB of these vegetation communities did not change significantly during this period, differences in the spectra and AGB still existed. Furthermore, we found that in some bottomlands, *A. selengensis* tended to grow mixed with *P. arundinacea*, which reduced the classification accuracy.

## Conclusions

In this study, the machine learning algorithm RF was used to estimate the AGB of the Poyang Lake wetlands and to obtain the AGB levels of four dominant vegetation communities on the basis of maps derived from classification images generated through an SID classifier in autumn from 2010 to 2016. The primary conclusions are as follows:

1. The coverage of each of the vegetation types is always changing as a result of water level fluctuations. From 2010 to 2016, *Carex cinerascens *had the largest distribution, with an average area of 569 km^2^. The distributions of *Phalaris arundinacea* and *Artemisia selengensis* were 226 and 200 km^2^, respectively. The smallest was *Miscanthus sacchariflorus*, with a distribution area of only 120 km^2^.
2. *M. sacchariflorus *had the highest AGB density, with an average value of 1.39 kg m^−2^; *C. cinerascens* and *A. selengensis* were almost at the same level with 1.26 and 1.28 kg m^−2^, respectively; and *P. arundinacea *had the lowest AGB density at 0.64 kg m^−2^.

## Data Availability Statement

All datasets generated for this study are included in the article/supplementary material.

## Author Contributions

RW conceived and designed the research. RW and PW wrote the manuscript. PW analyzed the data. XW designed the field work and performed the field investigations from 2010 to 2016. XY and XD performed the field survey and experiments.

## Funding

This study was supported by the Key Research Program of Chinese Academy of Sciences (No. KFZD-SW-318) and the National Natural Science Foundation of China (No. 41571107).

## Conflict of Interest

The authors declare that the research was conducted in the absence of any commercial or financial relationships that could be construed as a potential conflict of interest.
